# Hematene Nanoplatelets with Enhanced Visible Light Absorption; the Role of Aromatic Molecules

**DOI:** 10.3390/molecules29133115

**Published:** 2024-06-29

**Authors:** Georgios Alpochoritis, Argiris Kolokithas Ntoukas, Vasilios I. Georgakilas

**Affiliations:** 1 Department of Materials Science, University of Patras, 26504 Patras, Greece; up1074158@upnet.gr; 2 Department of Pharmacy, School of Health Sciences, University of Patras, 26504 Patras, Greece; kolokithas@upatras.gr

**Keywords:** hematene, water splitting, light absorptivity, 2D nanoplatelets

## Abstract

Hematite has been considered an important candidate for the development of an efficient photoelectrocatalytic water-splitting system. One of the most serious obstacles that limits the efficiency of hematite is low absorption capacity in visible light. Herein, we report the production of hematene nanoplatelets from hematite ore with yields of up to 60%, using a low-cost, sustainable method that is based on the ultrasonic treatment of hematite ore in a water solution of a series of organic aromatic compounds. We show that the chemisorption of molecules with increased aromaticity on the surface of hematene resulted in the significant improvement of its visible light absorptivity, with an increase of up to 200%. As a result, using a water solution of terephthalaldehyde as a liquid medium, hematite ore was exfoliated to hematene nanoplatelets with a yield of 40% and remarkable stability in water. Due to this, hematene was easily drop-casted on glass forming homogenous thin films with strong absorptivity in the visible region.

## 1. Introduction

Hematite (α-Fe_2_O_3_) is the most thermodynamically stable form of iron oxide, found in abundance on Earth and easily prepared in the laboratory. It has been extensively studied as an anodic material for photoelectrochemical (PEC) water splitting that is considered a great challenge for solar energy conversion [1,2]. Hematite has certain significant advantages and among others is a photochemically stable, non-toxic, environmentally friendly semiconductor with an energy bandgap of about 2 eV that suggests a highly promising theoretical maximum solar-to-energy efficiency of 15% [3]. However, experimental results have shown much lower values, although an extensive number of ideas have been explored in recent years such as metal doping; size, shape and surface area control; and hybrid or heterostructure fabrication [4,5,6]. The four main disadvantages of hematite that limit its efficiency focus on poor conductivity, short lifetime of photoelectrons due to recombination, slow water oxidation reaction, and poor light absorption capacity [1].

The development of nanostructured materials in the last few decades has offered new perspectives on semiconductors that can be used in hydrogen evolution reaction (HER) [7,8], photovoltaics [9,10] and photoelectrocatalytic water splitting [11]. Therefore, controlling the size, the shape and the surface morphology of nanostructured hematite could facilitate charge separation, provide short diffusion length for minority carriers and optimize the light absorption and energy bandgap, limiting the disadvantages of the bulk form [3,12]. In recent years, following the great success of graphene, a variety of 2D nanostructures have been prepared by the liquid phase exfoliation (LPE) method, from layered materials such as transition metal dichalcogenides and group VA monoelemental solids like black phosphorous and hexagonal boron nitride [13].

Hematite ores belong to the family of non van der Waals materials that, under certain treatments, can be converted to 2D nanostructured analogs, such as hematene, which exhibit interesting optoelectronic properties and introduce novel expectations in various applications [14]. Two-dimensional hematene nanoplatelets have up to now been studied as a support in catalysis and in nonlinear optics, photocatalysis and photoelectrocatalytic water splitting [15,16,17,18,19,20,21].

Specularite, a lamellar hematite ore, has been exfoliated under sonication in organic solvents to very thin nanoplatelets that can be used as an alternative low-cost iron oxide nanostructure in catalysis [15]. Although polar organic solvents like dimethylformamide or N-methyl pyrrolidone are mainly used in the LPE process, water remains the most abundant, sustainable and environmentally friendly material and therefore the most interesting and desirable solvent [16]. In the literature, there are only a few examples of water being used as the solvent of the LPE of iron oxide materials. Specularite ore has been exfoliated to hematene nanosheets by LPE in water with a prolonged sonication of 48 h [20]. The sonochemical treatment has also been used for the exfoliation of synthetic hematite nanopowder in the form of flakes, which showed much higher photoelectrochemical efficiency [21].

Recently, we have shown that iron oxide ores, such as hematite and magnetite can be exfoliated in an aqueous solution of melamine under mild sonication. The isolated nanoplatelets of hematene showed interesting nonlinear optical properties [17].

Following this idea, we describe here the formation of hematene nanoplatelets with highly visible light absorption (VLA) resulting from the treatment of hematite ore with mild sonication in aqueous solutions of a variety of organic aromatic compounds. Depending on the organic compound used, the resulting hematene nanoplatelets exhibited high VLA, and excellent fabrication efficiency. The aromatic compounds investigated here have some common characteristics, such as low to mild solubility in water, planar shape, presence of oxygen (O) or nitrogen (N) atoms and aromaticity. The aim of the present work was to study the role of organic molecules in shaping some important characteristics of the hematene nanoplatelets in terms of the photoelectrocatalysis of water splitting, such as stability and homogeneous dispersion in water, VLA and the energy bandgap.

## 2. Results and Discussion

**Figure 1:**
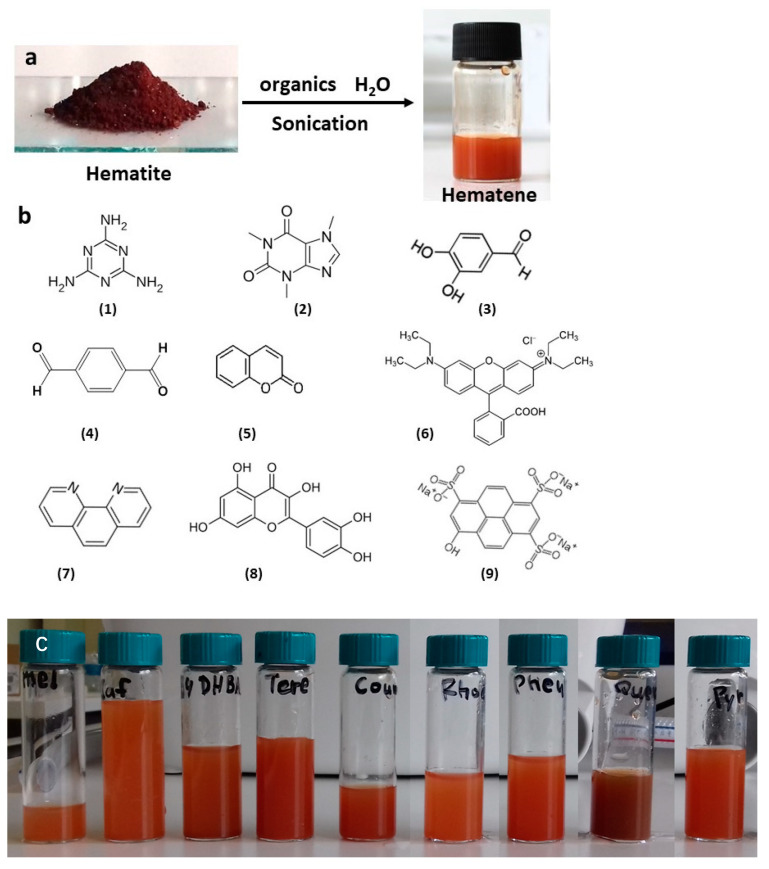
(**a**) Schematic representation of the exfoliation procedure; (**b**) the organic compounds that were used in water solution: melamine (**1**), caffeine (**2**), 3,4 dihydroxy benzaldehyde (**3**), terephthalaldehyde (**4**), coumarin (**5**), rhodamine b (**6**), phenanthroline (**7**), quercetin (**8**) and pyranine (**9**); (**c**) the dispersion of the hematene products in water.

Hematite ore is red brown in color and its optical absorption spectrum extends from the near-infrared to the ultraviolet region with maximum absorption in the 400 nm region. The raw material used in this work was natural ferroxide, hematite. It is associated with Fe-skarns and occurs as patches that replace magnetite due to supergene oxidation. A certain amount of hematite ore was dispersed in a solution of the organic compound in water and the mixture was sonicated in a bath for an hour. The non-exfoliated material was removed after precipitation and the dispersed material was isolated by filtration and washed to remove the organics. The organic compounds that were used in this work, as well as the dispersed-in-water hematene products, are shown in Figure 1.

**Figure 2:**
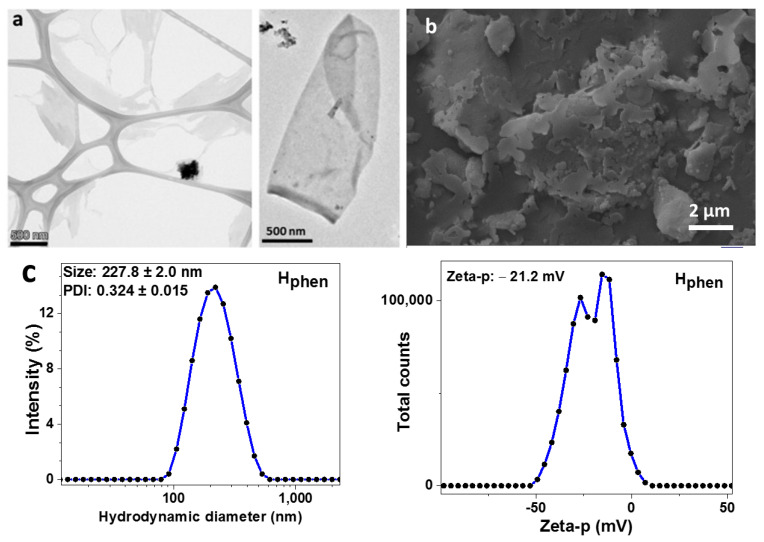
(**a**) TEM and (**b**) SEM images of hematene nanosheets from H_mel_ product. (**c**) Hydrodynamic diameter and zeta potential of H_phen_ measured by DLS.

The liquid phase exfoliation (LPE) yield ranged between 30 and 60%. The two amine-rich compounds, melamine and caffeine, showed the highest yield close to 60%, the aldehydes, 3,4 DHBA and terephthalaldehyde, showed moderate yields around 40% and the rest, oxygen-rich compounds, showed the lowest but remarkable yields of about 30%. The compounds used in the present work, except for pyranine, have rather low solubility in water. Recently, our group has shown that poorly soluble organic compounds in water favor graphite exfoliation and the production of few-layer graphene nanosheets [22]. However, in terms of the ultrasonic treatment of hematite, the solubilities of organic compounds in water, do not have a direct impact on the performance of this treatment. The LPE of hematite in the presence of organics produces very thin nanoplatelets, with a thickness of much less than 5 nm [17] and a lateral size from 0.5 to a few μm (see Figure 2a,b). The dynamic light scattering (DLS) measurements (see Table 1 and Figure S1) showed that the prepared hematene nanoplatelets have similar hydrodynamic diameters (except that of H_rhod_) ranging between 230 and 360 nm, indicating that the role of organic molecules in terms of the structural characteristics of the nanoplatelets is rather limited.

**Figure 3:**
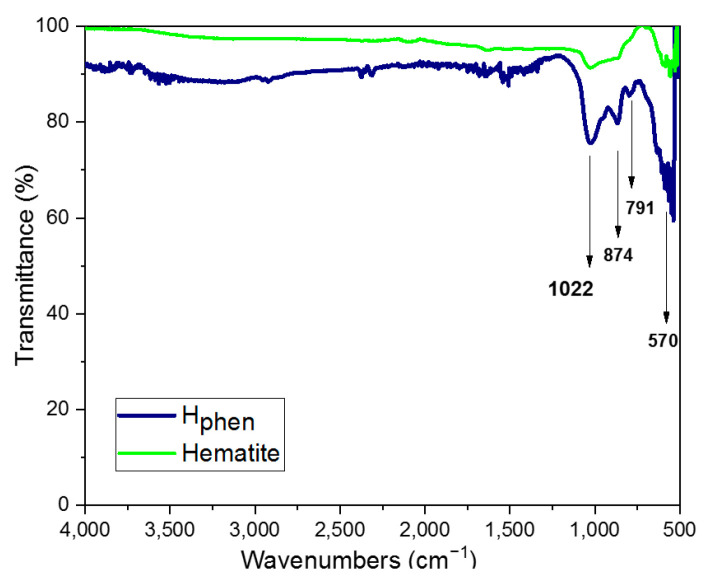
FTIR spectra of hematite ore and the product H_phen_.

The FTIR spectra of the hematene products and hematite showed similar bands (see Figure 3). The peaks at 874 and 570 cm^−1^ are characteristic Fe-O vibrations. The remaining two peaks at 1022 and 791 cm^−1^ are attributed to stretching vibrations associated to goethite impurities of the starting material [23]. The X-ray diffraction (XRD) patterns of the hematene nanoplatelets include peaks corresponding to the main lattice planes of bulk hematite. However, the peaks of the nanoplatelets are much broader, as expected from the nanoscale dimensions (see Figure S2 and Table S1).

The main property of hematene is its characteristic absorption in the UV–Vis area, which makes it a very promising semiconductor, suitable for photoelectrochemical water splitting.

**Figure 4:**
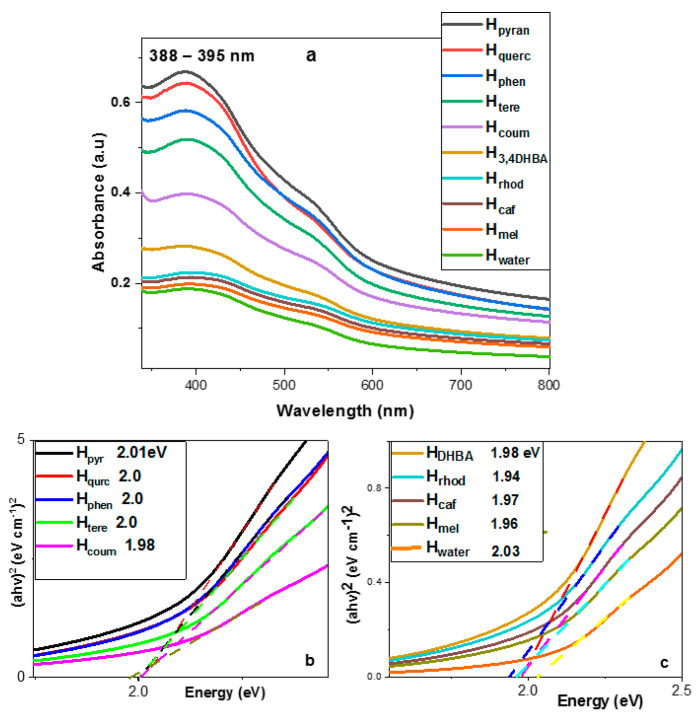
(**a**) UV–Vis absorption spectra of hematene samples in water dispersion. (**b**,**c**) Tauc plot graph for direct transitions optical band gaps calculated by extrapolation of the linear part of (ahv)^2^ versus energy.

UV–Vis spectra comparison. The samples were diluted with water to a final concentration of 0.09 g L^−1^. The absorption spectra of the samples are presented in Figure 4a. The absorption of hematene starts in the near-infrared region, continues to increase slightly in the visible and finally forms an intense broad band with λ_max_ at 388–395 nm and a broad shoulder around 530–550 nm attributed to ligand-to-metal charge transfer transitions and pair excitations of two adjacent Fe^3+^ cations occupying adjacent sites, respectively. Based on experimental data, the λ_max_ position depends on the thickness of the nanoplatelets due to quantum confinement. The decrease in the thickness of the hematene nanoplatelets is associated with a blue shift of the λ_max_ [17,23]. The almost constant value of λ_max_, here, suggests that the thickness of the different hematene nanoplatelets is very similar.

Considering the concentration of the hematene samples (C = 0.09 g L^−1^) and the absorbance at 380–400 nm, the extinction coefficient of the hematene products was estimated (see Table 2). Based on the Tauc plot method, the main direct bandgaps of the hematene samples were also estimated (see Table 2 and Figure 4b,c). The absorption efficiency of the hematene samples in aqueous dispersion in the visible light region showed the highest values with H_pyran_, H_querc_ and H_phen_ and the lowest with H_mel_ and H_caf_. The most effective product, H_pyran_, has about 4 times higher VLA (α_550_ = 0.35) than hematene produced in pure water, H_water_ (α_550_ = 0.09). The energy bandgaps of the hematene products showed a similar trend with the absorbance, although the area that ranges is very narrow.

**Figure 5:**
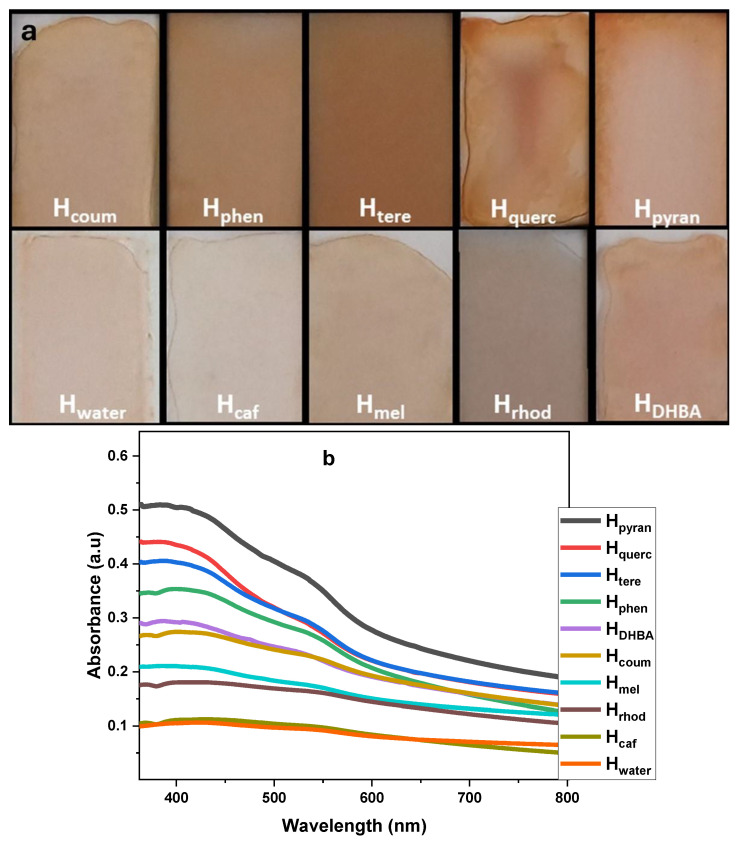
(**a**) Thin films of hematene deposited on glass slides; (**b**) absorption spectra of hematene films.

A total of 200 μg of the hematene samples was deposited on glass slides by simple drop-casting of aqueous dispersions and air-dried to form thin films. Most of the samples formed homogenous films. In the H_pyran_ sample, a coffee ring with a high concentration of hematene at the periphery was formed, while in the H_querc_ sample an aggregate was formed in the middle (see Figure 5a). Subsequently, the air-dried hematene films were then studied by UV–Vis spectroscopy (see Figure 5b). VLA was recorded in different regions of the covered surface to examine the homogeneity of the films.

**Figure 6:**
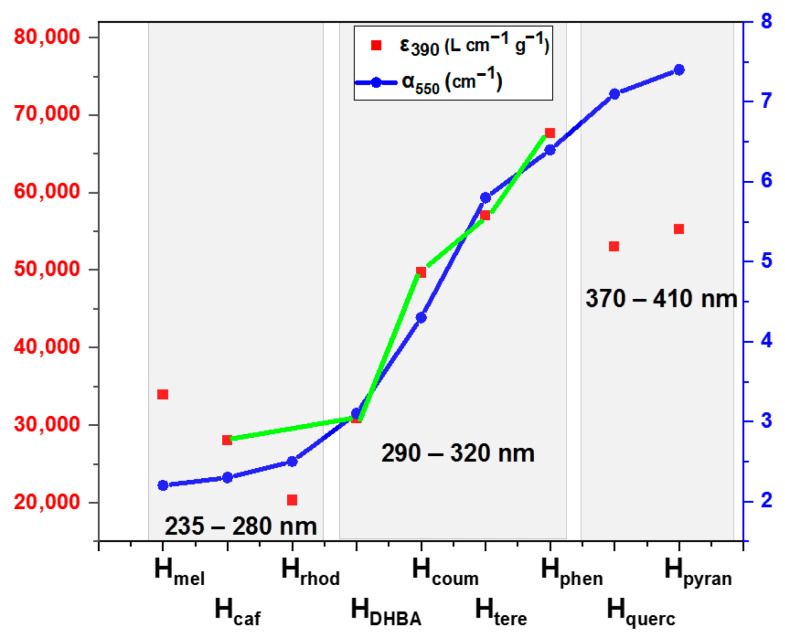
The extinction coefficient ε_390_, the absorption coefficient α_550_ and the yield of the preparation procedure of the hematene products are shown. In the gray highlighted areas, the area of the λ_max_ of the absorption spectra of the involved aromatic compounds is shown.

The absorption coefficient α was calculated using the equation α = 2.30 × A/thickness. The average thickness of the samples was estimated as 0.17 μm. The absorption coefficients, α, of the hematene films are presented in Table 2. It was noted that the extinction coefficient (measured in water dispersion) and the absorption coefficient (measured in films) showed a similar trend, in terms of increasing absorptivity, except for H_pyran_ and H_querc_ which are probably affected by the inhomogeneity of the films (see Figure 6). The size and shape of hematite nanostructures have been reported among the factors affecting their absorptivity and energy bandgap [12,24,25]. However, here, the 2D shape is the same for all the hematene nanoplatelets and their size and thickness do not differ significantly, as shown by DLS measurements and the position of λ_max_.

On the other hand, the experimental results showed that the absorptivity of the final hematene nanoplatelets is in accordance with the aromaticity of the molecules interacting with the hematene nanoplatelets during exfoliation. The increased aromaticity of the organic molecules is associated with the red shift of their UV–Vis absorption bands due to the stabilization of π* and the lower energy of π,π* transition. Therefore, the interaction of hematene with pyranine and quercetin, which have a higher aromatic character as indicated by their absorption spectra, is associated with the most absorptive nanoplatelets H_pyran_ and H_querc_. On the other hand, the less aromatic caffeine and melamine, lead to the formation of the less absorbing H_caf_ and H_mel_ nanoplatelets. Finally, molecules with moderate aromaticity have contributed to the formation of hematene products with medium-to-high absorptivity (see Figure 6).

H_phen_ showed the highest VLA capacity in solution and in film, but it was produced with a medium yield. H_mel_ was produced with the highest yield, but its absorption capacity was the lowest. The solution of terephthalaldehyde in water was the optimum case to produce hematene, H_tere_, with a high absorption capacity and a very promising yield.

**Figure 7:**
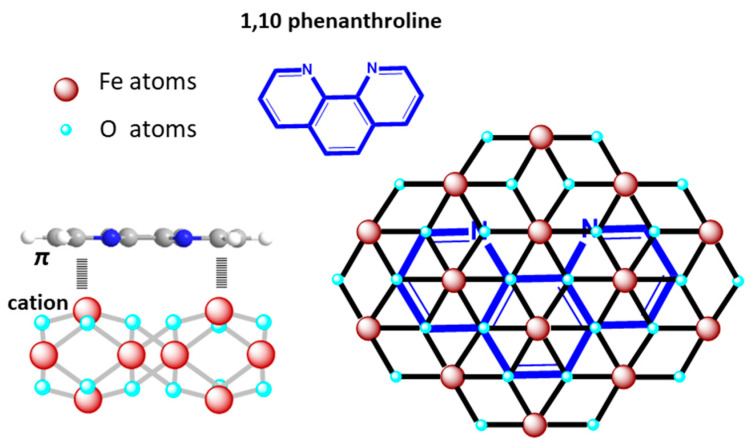
The π-cation interactions between hematene and the aromatic molecule, 1,10 phenanthroline.

Here, the role of organic molecules during the formation of hematene nanoplatelets in water is determined by its interaction with the surface of the hematene. Although the nature of those interactions has not yet been studied in detail, cation–π interactions of aromatic compounds with mineral surfaces have been reasonably suggested, as they are often identified in similar natural systems [26,27]. The planarity of the aromatic molecules favors these interactions and the position of the molecules parallel to the hematene surface (see Figure 7) is the most energetically stable, taking into consideration the DFT study of the adsorption of benzene on hematite surfaces by Dzade et al. [26].

Εxamining the UV–Vis spectra of the hematene products at shorter wavelengths (e.g., 200–400 nm), weak absorption bands were detected, indicating the presence of small amounts of organic molecules on the surface of hematene nanoplatelets after washing (Figure S3). Based on this observation, it is suggested that, here, the cation–π interactions of the hematene surface with aromatic molecules facilitate the transition of the electrons of the semiconductor to the conduction band and significantly increase the VLA of the hematene nanoplatelets, offering a great advantage for their use in photoelectrocatalytic water-splitting systems. The effect on the water-splitting efficiency of organically modified hematene nanoplatelets due to increased absorptivity is likely to be mitigated by the restriction of the catalytic centers on the surface of the hematene, due to the presence of the organic molecules. However, spectroscopic evidence, in particular the lack of significant signals of organic material on the hematene FTIR spectra, suggests that the presence of these molecules on the surface is limited in contrast with their impact.

## 3. Materials and Methods

Materials. The raw material is a natural ferroxide, hematite, from the mining areas in Selero, Xanthi (North Greece). The following were used as received: Melamine, Terephthalaldehyde (TCI, Glendenning, Australia), 1,10 Phenanthroline and Rhodamine B (Acros Organics, Wakefield, MA, USA); Quercetin (Cayman Chemical, Ann Arbor, MI, USA); 3,4 Dihydroxybenzaldehyde and Pyranine (Sigma Aldrich, St. Louis, MO, USA); Caffeine and Coumarin (Thermo Fisher Scientific, Waltham, MA, USA).

Preparation method. A total of 20 mg of powder hematite was mixed with a solution of 100 mg of the organic compound (with 50 g pyranine) in 40 mL of deionized water, and the mixture was sonicated for 1 h in bath sonication. The remaining hematite was left to precipitate for 1 h and the supernatant was isolated and centrifuged for 30 min at 10,000 rpm to separate the solid hematene products from the organic material. The product was then redispersed in water, filtered (nylon membrane 0.45 μm), washed with water and dried in air. The thin films were formed by the drop-casting of 200 μg of the hematite samples dispersed in water and air-dried.

Characterization. XRD patterns was conducted with a D8 Advance Bruker diffractometer (Bruker AXS, Karlsruhe, Germany) using a CuKa (lD 1.5418) radiation source (40 kV, 40 mA) and a secondary beam graphite monochromator. The laser power was 1.082 mV. The optical spectra were recorded in water dispersion with a Shimadzu UV-1900 (Shimadzu, Duisburg, Germany). FTIR spectra were obtained with an ATR technique on a Fourier transform spectrometer (IRTracer-100, Shimadzu Europa GmbH, Duisburg, Germany). TEM images were obtained by a JEOL (Tokyo, Japan), JEM-2100 instrument operating at 200 kV.

## 4. Conclusions

The adsorption of aromatic organic compounds on the surface of hematene nanosheets during LPE of hematite in aqueous solutions results in a significant enhancement of the VLA of the 2D products. In fact, the higher the aromatic character of the organic molecules, the higher the VLA. Using organic aromatic molecules such as terephthalaldehyde, the VLA was increased up to 200% with a satisfactory yield. The increased VLA of hematene could contribute to the fabrication of electrodes for photoelectrochemical water splitting with satisfactory performance.

## Figures and Tables

**Table 1:** The hydrodynamic diameter and zeta potential of the products in water.

	Hydrodynamic Diameter (nm)	PDI	Zeta Potential (mV)
H_mel_	310.9 ± 25.9	0.42 ± 0.07	−15.9
H_caf_	362.7 ± 5.9	0.41 ± 0.03	−19.1
H_DHBA_	272.9 ± 4.3	0.47 ± 0.01	−20.5
H_Tere_	294.3 ± 3.6	0.42 ± 0.03	−15.7
H_coum_	258.1 ± 8.9	0.37 ± 0.04	−20.1
H_rhod_	566.8 ± 15.2	0.65 ± 0.05	−14.9
H_phen_	227.8 ± 2.0	0.32 ± 0.01	−21.2
H_querc_	297.4 ± 2.9	0.41 ± 0.06	−18.9
H_pyran_	254.6 ± 12.3	0.44 ± 0.04	−19.3

**Table 2:** The absorbance at the λ_max_ of the dispersion of hematene products in water and the estimated extinction coefficient, ε, and energy bandgap. The last two columns show the mean value of the absorption coefficient, α, of the hematene samples deposited as thin films and the relative standard deviation (RSD).

Samples	Absorbance λ_max_	ε_390_ L cm^−1^ g^−1^	Bandgap eV	α (550 nm) cm^−1^	RSD %
H_mel_	0.20 (393)	2.2	1.97	33,935	6.1
H_caf_	0.21 (393)	2.3	1.98	27,998	6.7
H_rhod_	0.23 (395)	2.5	1.94	20,305	5.6
H_DHBA_	0.28 (387)	3.1	1.98	30,907	0.8
H_coum_	0.39 (389)	4.3	1.98	49,661	6.3
H_Tere_	0.52 (390)	5.8	2.0	56,998	1.1
H_phen_	0.58 (387)	6.4	2.0	67,638	2.6
H_querc_	0.64 (387)	7.1	2.0	53,069	12.8
H_pyran_	0.67 (386)	7.4	2.01	55,285	6.5

## Data Availability

The data presented in this study are available in article and Supplementary Materials.

## Supplementary Materials

The following supporting information can be downloaded at: https://www.mdpi.com/article/10.3390/molecules29133115/s1, Figure S1: DLS and zeta potential measurements of the hematene products; Figure S2: XRD patterns of the hematene products; Figure S3: UV–Vis spectra of the hematene products and the organic aromatic compounds that were chemisorbed on the hematene surface after sonication; Table S1: The characteristic signals of the SRD patterns of the hematene products.
