# Supplementary material for: Hematene Nanoplatelets with Enhanced Visible Light Absorption; the Role of Aromatic Molecules
Source: Molecules. 2024 Jun 29;29(13):3115. doi: 10.3390/molecules29133115 (PMC11243378; doi:10.3390/molecules29133115)
Supplement: Supplementary file 1 [file molecules-29-03115-s001.zip › molecules-3040912-supplementary.pdf]

Hematene nanoplatelets with enhanced visible light absorption; the role of aromatic molecules

Georgios Alpochoritis,[a] Argyris Kolokythas Ntoukas[b] and Vasilios Georgakilas* [α]

Supplementary Material

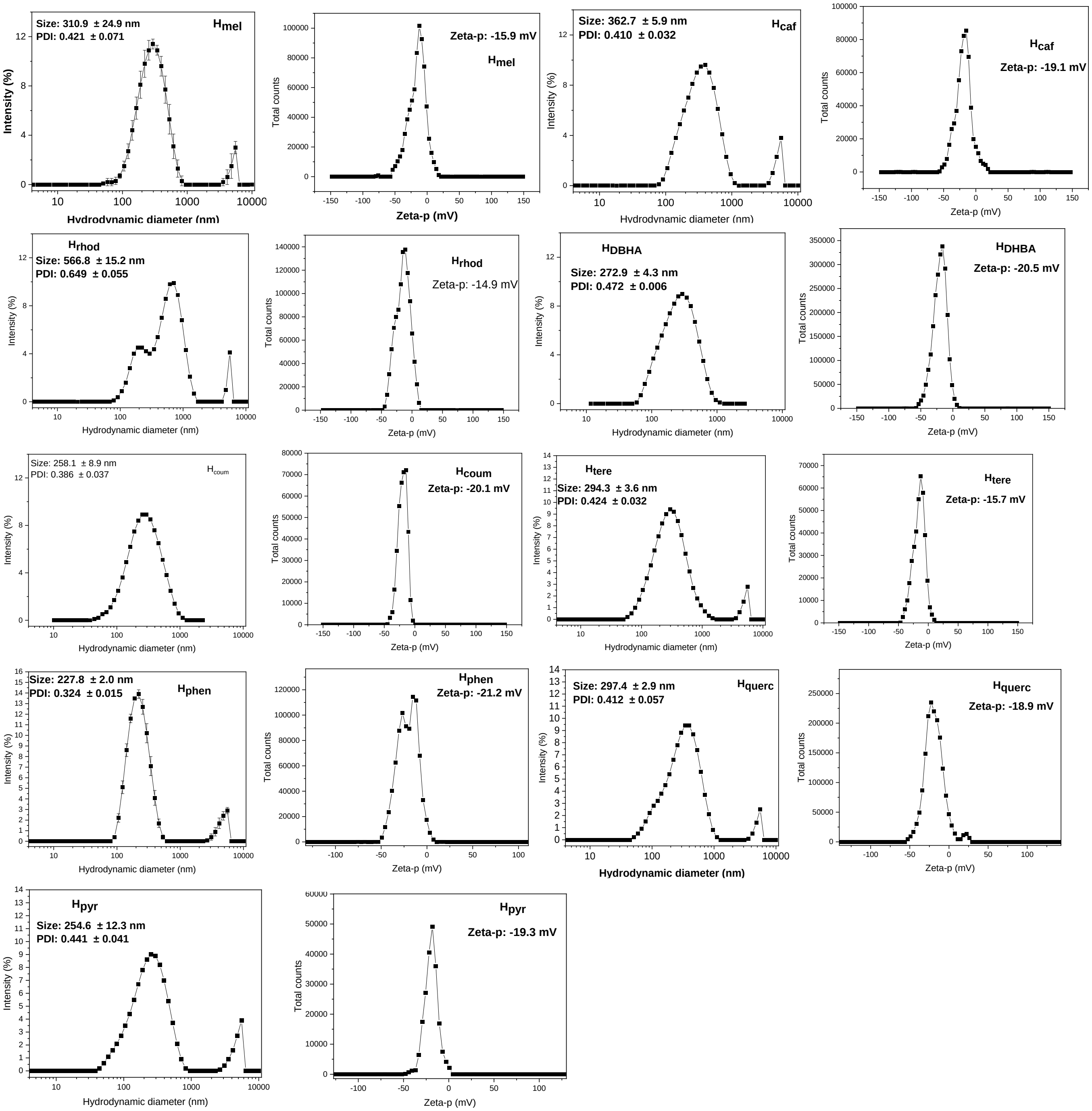

Figure S1. DLS and zeta potential measurements of the hematene products.

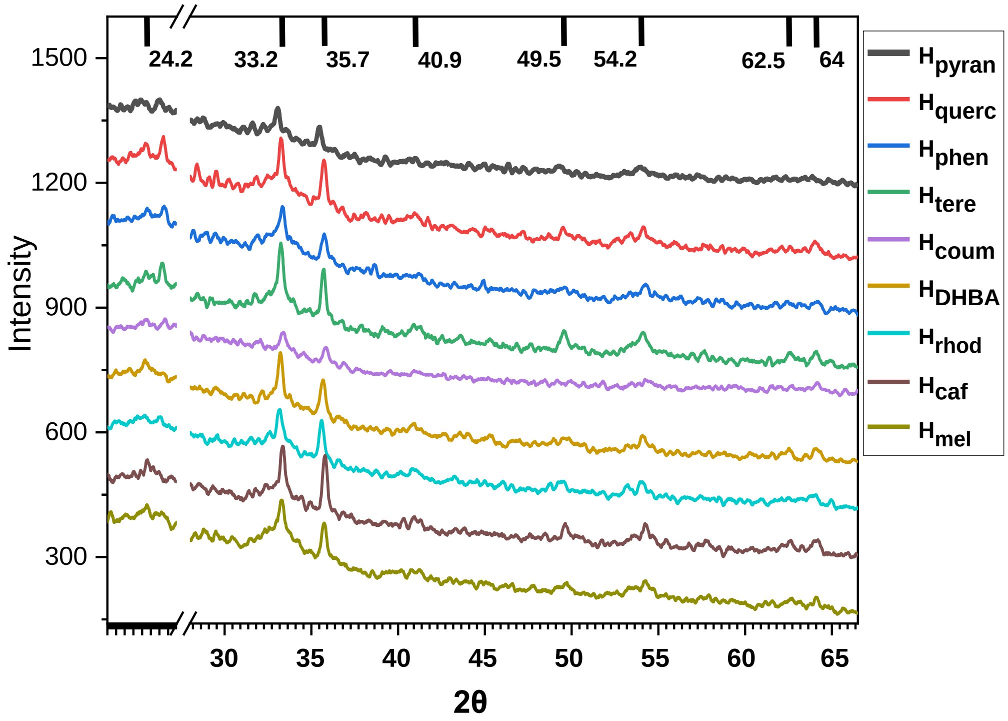

Figure S2. XRD diffraction patterns of the hematene products.

Table S1. The characteristic signals of the SRD diffraction patterns of the hematene products.

	(0,12)	**(104)**	**(110)**	(113)	(0,24)	(116)	(018)	(214)	(030)		FWHM
Hematite	24.2	33.27	35.7	40.9	49.5	54.1	57.6	62.5	64.14	72.1	0.14
H_{pyran}	24.1	33	35.5		49.3	54					0.42
H_{auer}	24.3	33.2	35.7	41	49.5	54			64		0.2
H_{phen}	24.1	33.3	35.8			54.3					0.34
H_{tere}		33.18	35.65	40.9	49.7	54.1	57.7	62.6	64		0.27
H_{coum}	24.3	33.3	35.8	41		54			64.2		0.36
H_{DHBA}	24.3	33.2	35.6	40.9	49.6	54		62.5	64		0.34
H_{rhod}		33.19	35.66	41	49.5	54.2			64		0.32
H_{caf}	24.3	33.3	35.8	41	49.6	54.2		62.4	64.1		0.31
H_{mel}	24.3	33.3	35.7	41	49.7	54.2	57.7	62.7	64.2		0.35

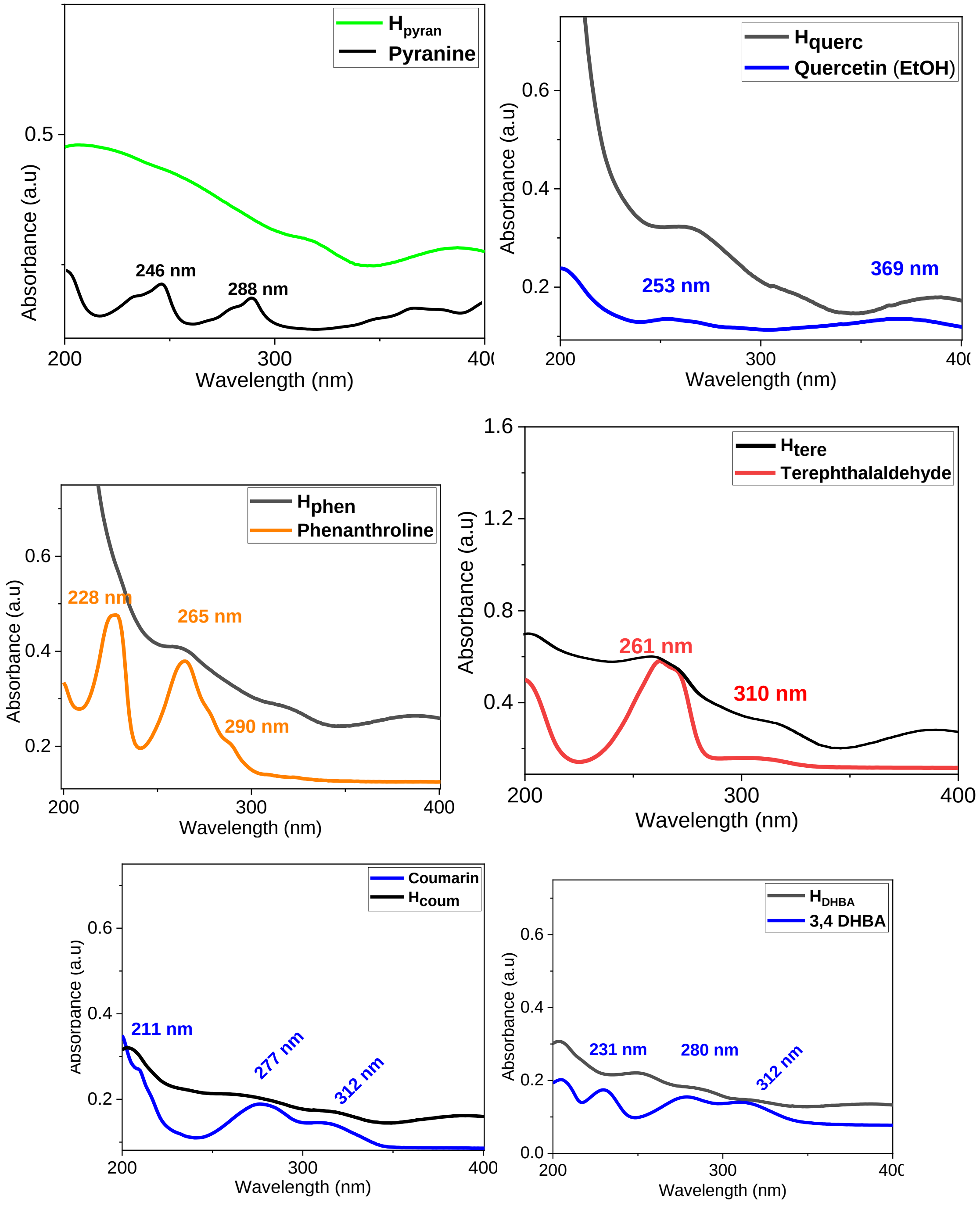
Hpyran
Pyranine
246 nm
288 nm
Absorbance (a.u)
Wavelength (nm)
Hquerc
Quercetin (EtOH)
253 nm
369 nm
Hphen
Phenanthroline
228 nm
265 nm
290 nm
Htere
Terephthalaldehyde
261 nm
310 nm
Coumarin
Hcoum
211 nm
277 nm
312 nm
HDHBA
3,4 DHBA
231 nm
280 nm
312 nm

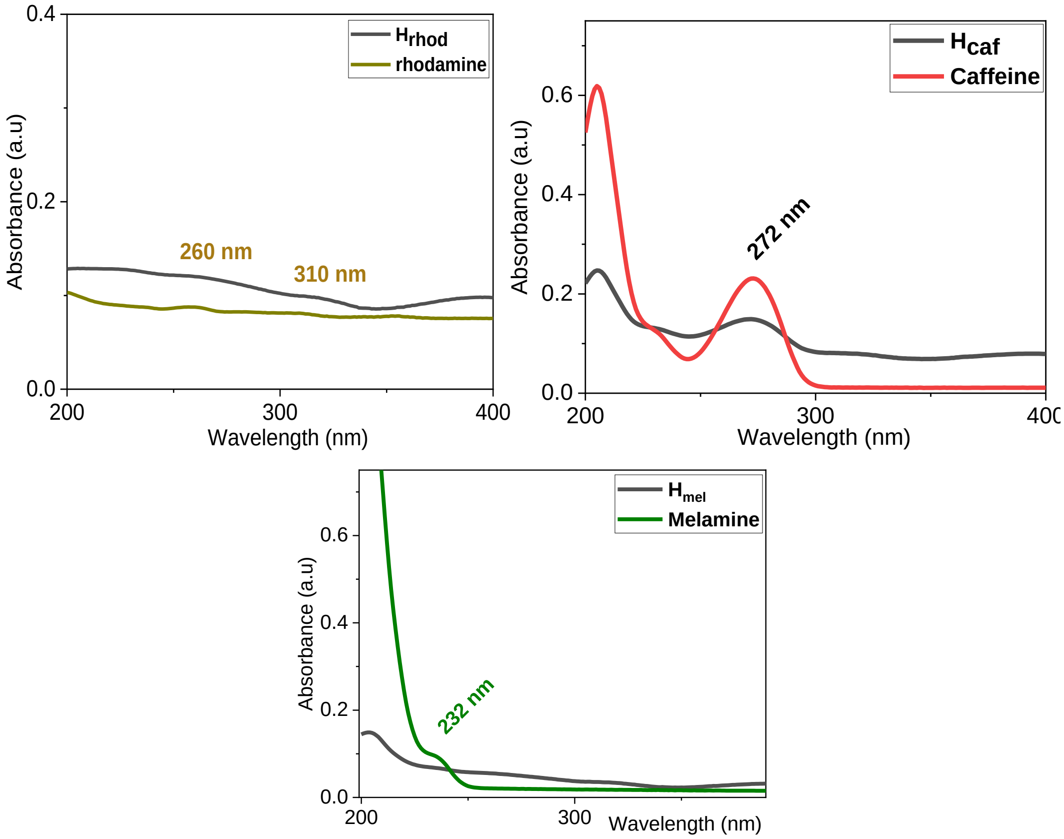

Figure S3. UV-Vis spectra of the hematene products and the organic aromatic compounds that were chemisorbed on the hematene surface after sonication.
